# Carboxymethylated *Poria cocos* Polysaccharides Mitigate Cisplatin‐Induced Hepatorenal Injury by Activating the PLC‐PKC‐Nrf2 Signaling Pathway

**DOI:** 10.1002/fsn3.72342

**Published:** 2026-09-05

**Authors:** Liang He, Shuo Chen, Baoming Wu, Jiong Gu, Conghan Li, Xianfan Du, Jun Chu, Min Yang, Hui Hou

**Affiliations:** ^1^ Department of General Surgery The Second Affiliated Hospital of Anhui Medical University Hefei Anhui China; ^2^ Key Laboratory of Xin'an Medicine, Ministry of Education Anhui University of Chinese Medicine Hefei Anhui China; ^3^ Inflammation and Immune Mediated Diseases Laboratory of Anhui Province Anhui Medical University Hefei Anhui China; ^4^ The 2nd Department of Intensive Care Unit The Second Affiliated Hospital of Anhui Medical University Hefei China

**Keywords:** carboxymethylated *Poria cocos* polysaccharides, cisplatin, hepatorenal injury, Nrf2, PKC

## Abstract

Cisplatin, a commonly used chemotherapy drug, inevitably causes damage to the liver and kidneys while exerting its anti‐tumor effects. Carboxymethylated *Poria cocos* polysaccharides (CMP) have anti‐inflammatory, antioxidant, and organ‐protective properties, and also exhibit significant anti‐tumor activity. Therefore, this study aims to investigate the effects of CMP on cisplatin‐induced hepatorenal damage in mice. The results showed that CMP treatment significantly mitigated abnormalities in hepatorenal function indicators and tissue damage induced by cisplatin. Furthermore, CMP significantly suppressed the increase in levels of inflammatory factors in peripheral and hepatorenal tissues, as well as the infiltration of immune cells induced by cisplatin. Additionally, CMP significantly enhanced the antioxidant capacity of hepatorenal tissues and inhibited oxidative damage caused by cisplatin. In vitro, CMP have also been shown to protect renal tubular epithelial cells and hepatocytes from cisplatin‐induced damage. Mechanistically, our studies indicated that CMP activate Nrf2 through a phosphorylation pathway that is independent of Keap1 and reliant on PLC‐PKC signaling. In conclusion, our findings suggest that CMP has the potential to serve as an adjunctive agent in cisplatin chemotherapy to protect the kidneys and liver from damage.

## Graphical abstract

CMP protects against cisplatin‐induced hepatorenal damage by suppressing inflammation and oxidative stress. CMP reduces serum ALT, AST, BUN, and Cr levels, ameliorates tissue pathological injury, inhibits pro‐inflammatory cytokine production and immune cell infiltration, and restores cellular antioxidant capacity. Notably, CMP triggers Keap1‐independent Nrf2 activation through the PLC‐PKC pathway: CMP‐activated PLC generates DAG to stimulate PKC, which phosphorylates Nrf2, facilitates its nuclear translocation, and induces downstream antioxidant genes (HO‐1, NQO1, GPX4, and SOD1) to exert hepatorenal protection.

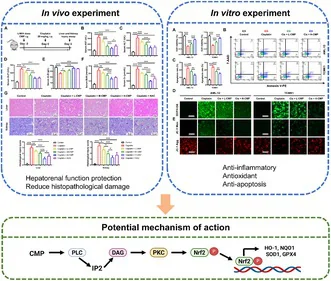

## 1 Introduction

Cisplatin is a widely utilized chemotherapeutic agent known for its extensive anti‐tumor properties (Dasari and Tchounwou 2014). Its primary mechanism of action involves the interference with DNA replication and repair processes within cancer cells, ultimately inhibiting tumor cell proliferation (Ghosh 2019). Intravenous infusion of cisplatin effectively targets tumor cells throughout the body; however, it also poses a risk of damaging healthy cells, which may lead to adverse effects such as hepatorenal toxicity, nausea, vomiting, and bone marrow suppression (Elmorsy et al. 2024). After cisplatin accumulates in liver and kidney tissues, it damages mitochondria and induces an excessive burst of ROS, inhibiting the Nrf2 antioxidant system and causing oxidative damage; subsequently, it activates the NF‐κB and MAPK inflammatory pathways, driving the release of pro‐inflammatory factors, ultimately mediating parenchymal cell apoptosis and organ injury (Santabarbara et al. 2016).

Several strategies have been implemented in clinical practice or are currently under investigation to reduce the toxicity of cisplatin, enhance patient tolerance, and improve overall quality of life. These strategies include the administration of protective chemotherapeutic agents, such as amifostine, as well as the use of hydration therapy and diuretics (Agrawal and Jain 2025; Markman 1998; Sikking et al. 2024). Additionally, the adoption of split‐dose cisplatin has been explored (Nakane et al. 2025), but this approach may extend the duration of treatment and potentially affect its efficacy (Elmorsy et al. 2024). Recent studies have indicated that bioactive components extracted from traditional Chinese medicine not only exhibit significant anti‐tumor properties but also possess the ability to protect organs from chemotherapy‐induced damage while simultaneously addressing resistance to both radiotherapy and chemotherapy (Fang et al. 2021; Xu et al. 2023; Zhang et al. 2024). Consequently, the exploration of effective extracts or individual components from traditional Chinese medicine as a means to mitigate the side effects of cisplatin represents a promising area for research.

*Poria cocos* (Schw.) Wolf, a medicinal fungus, and its dried sclerotia are utilized in traditional Chinese medicine and possess a range of pharmacological functions, such as anti‐tumor, hepatoprotective, anti‐aging, diuretic, anti‐inflammatory, immune‐enhancing, and lipid‐lowering effects (Lei et al. 2025). *Poria cocos* polysaccharides (PCP) represent a primary active constituent, accounting for 70%–90% of the sclerotia's dry weight (Ng et al. 2024), exhibit various medicinal activities, including immunoregulatory, anti‐cancer, anti‐inflammatory, and antioxidant (Deng and Huang 2024). However, PCP has poor water solubility and a high molecular weight, which limit its bioavailability. Carboxymethylation, the most widely used chemical modification method for polysaccharides, significantly improves water solubility and alters chain conformation by introducing carboxymethyl groups (—CH_2_COOH). Importantly, carboxymethylated *Poria cocos* polysaccharides (CMP) not only retain their original antioxidant and anti‐inflammatory activities but also significantly enhance their anti‐tumor activity (Liu et al. 2019; Wang et al. 2015). Additionally, our previous research has demonstrated that CMP have significant anti‐inflammatory properties and effectively inhibit septic kidney injury in mouse models (Zhang, Chen, et al. 2025).

Numerous studies have established that PCP exhibits immunomodulatory properties, which may enhance the body's anti‐tumor immune response by modulating the functions of immune cells (Li et al. 2023; Lv et al. 2024; Yu et al. 2025). Furthermore, recent research has demonstrated that compounds derived from *Poria cocos*, when administered in combination with carboplatin or paclitaxel, can significantly improve the tumor response rate in patients and reduce the side effects of chemotherapy (Peng et al. 2022). Several studies have indicated that PCP may enhance intestinal barrier integrity and diminish chemotherapy‐induced intestinal toxicity by influencing the gut microbiota (Yin et al. 2022; Zou et al. 2021). Therefore, PCP holds potential as an adjunctive treatment in chemotherapy, particularly in alleviating its side effects. However, the impact of PCP on cisplatin‐induced hepatorenal injury remain unclear. This study aims to examine the impact of PCP and elucidate its underlying molecular mechanisms on hepatorenal damage induced by cisplatin.

## 2 Material and Methods

### 2.1 Grouping and Modeling of Mice

C57BL/6J mice (male, 8 weeks old and weighing 24 ± 2 g) were procured from Liaoning Changsheng Biotechnology Co. Ltd., with animal production license number SCXK (Liao) 2020‐0001. The mice were housed at the Experimental Animal Center of Anhui University of Traditional Chinese Medicine under controlled environmental conditions, including a temperature range of 23°C–25°C, relative humidity of 50% ± 5%, and a 12‐h light/dark cycle, with unrestricted access to water and food. The experimental protocol involving animals for this study received approval from the Animal Ethics Committee of Anhui University of Traditional Chinese Medicine (Number: AHUCM‐mouse‐2024235).

*Poria cocos* sclerotia were purchased from Anhui Xiehe Cheng Pharmaceutical Co. Ltd. (Bozhou, Anhui, China). CMP were prepared as previously described (Zhang, Chen, et al. 2025). Briefly, crude polysaccharides were obtained after extraction with NaOH solution (liquid‐to‐solid ratio 1:10). In an 80% ethanol aqueous solution, the reaction is carried out with the following molar ratio of substances: polysaccharide: NaOH: chloroacetic acid = 1: 3.5: 1.75. NaOH was added in two portions, followed by chloroacetic acid for substitution at 60°C for 6 h. The pH was neutralized with glacial acetic acid. The precipitate was re‐dissolved in water, precipitated with 75% ethanol, and lyophilized to obtain the crude polysaccharide.

Cisplatin (HY‐17394), NAC (HY‐B0215), and ML385 (HY‐100523) were purchased from MCE (USA). CMP and cisplatin solutions were prepared by dissolving them in normal saline. The ML385 solution was prepared by dissolving it in a 1:9 ratio of DMSO to corn oil. Mice were administered a single intraperitoneal injection of cisplatin at a dosage of 20 mg/kg to construct the model (Yu et al. 2026), whereas the control group was given an equivalent volume of normal saline. CMP were administered orally to the mice 3 days prior to the cisplatin injection. The CMP doses were selected based on our previous study (Zhang, Chen, et al. 2025). After euthanizing the mice, serum, liver, and kidney tissues were collected.

The mice were divided into six groups (*n* = 6 per group) based on weight matching. The experimental design comprised several groups: a control group, a cisplatin‐only group, a cisplatin combined with low‐dose CMP (L‐CMP, 100 mg/kg) group, a cisplatin combined with medium‐dose CMP (M‐CMP, 200 mg/kg) group, a cisplatin combined with high‐dose CMP (H‐CMP, 400 mg/kg) group, and a cisplatin combined with N‐acetylcysteine (NAC) group, which served as the positive control. These groups were used for in vivo functional validation of CMP.

Furthermore, the mice were allocated into five distinct groups (*n* = 6 per group) for in vivo validation of the Nrf2 signaling‐dependent protective effect of CMP: a control group, an ML385‐treated group, a cisplatin‐treated group, a group receiving both cisplatin and CMP, and a group treated with cisplatin, CMP, and ML385.

### 2.2 Assessment of Hepatorenal Function

The assessment of liver and kidney function is conducted through the measurement of serum levels of aspartate aminotransferase (AST), alkaline phosphatase (ALP) activity, and alanine transaminase (ALT), in addition to evaluating levels of creatinine (Cr), albumin (ALB), and blood urea nitrogen (BUN). BUN (ADS‐W‐N013) and Cr (ADS‐W‐FM034) assay kits were purchased from Jiangsu Aidisheng. Assay kits for AST (CN‐E‐BC‐K236‐M), ALT (CN‐E‐BC‐K235‐M), ALP (CN‐E‐BC‐K091‐M), and ALB (CN‐E‐EL‐M3032) were procured from Elabscience (Wuhan, China). The testing procedure should be conducted by kit manual. The results were analyzed using a microplate reader.

### 2.3 Histology

Fresh liver and kidney tissues were fixed in 4% paraformaldehyde overnight, followed by dehydration and paraffin embedding. Subsequently, the paraffin‐embedded hepatorenal specimens were sectioned at a thickness of 4 μm and stained using hematoxylin and eosin (H&E) staining solution (G1121, Solarbio, Beijing, China), and examined under a microscope. Histological scoring of H&E‐stained liver sections was performed using the modified histologic activity index (HAI), as described in a previous study (Feng et al. 2025). Four features were semi‐quantitatively graded as follows: 0, intact hepatocytes without evidence of degeneration or necrosis; 1, presence of minimal spotty necrosis accompanied by mild hepatocyte hydropic degeneration; 2, focal and patchy necrosis with infiltration of inflammatory cells; 3, multifocal confluent necrosis with abundant inflammatory cell presence; and 4, extensive submassive or panlobular necrosis. For kidney tissue damage, tubular injury was scored based on the percentage of affected tubules (0, none; 1, < 10%; 2, 10%–25%; 3, 26%–50%; 4, > 50%), evaluating tubular dilation, epithelial necrosis, cast formation, and interstitial edema, as described in a previous study (Zhang, Chen, et al. 2025). All scoring was performed by two independent pathologists masked to group allocation.

### 2.4 Assessment of Inflammatory Factors

The serum levels of interleukin‐6 (IL‐6), tumor necrosis factor alpha (TNF‐α), and interleukin‐1 beta (IL‐1β) were detected using commercial kits (NeoBioscience; Cat# EMC102a, EMC001b.96, and EMC004, respectively). MCP‐1 levels were measured using a commercial ELISA kit (NeoBioscience; Cat# EMC113). Following the completion of sample incubation, reaction, and color development as per the manufacturer's instructions, the results were recorded at 450 nm using a microplate reader.

### 2.5 Assessment of Antioxidant Enzyme and Oxidative Stress

Hepatorenal tissues were homogenized in pre‐cooled phosphate‐buffered saline (PBS) at a mass‐to‐volume ratio of 1:9, then centrifuged, and the supernatant was collected for analysis. Antioxidant enzymes, including glutathione catalase (CAT), peroxidase (GPX), and superoxide dismutase (SOD), were quantified utilizing assay kits obtained from KeyGEN BioTECH (Nanjing, China), with catalog numbers KGA7312‐100, KGA7301‐48, and KGA7310‐25, respectively. The parameters for oxidative stress, including malondialdehyde (MDA) and glutathione (GSH), were measured using the kits from Beyotime (Shanghai, China), with product numbers S0131 and S0053, respectively. After completing sample incubation, reaction, and color development according to the provided instructions, the results were recorded using a microplate reader.

### 2.6 Cell Culture

The AML12 (alpha mouse liver 12) and TCMK1 (transformed C3H mouse kidney‐1) cell lines were purchased from Fenghui Biotechnology (Changsha, China). The AML12 cells were maintained in DMEM/F12 medium containing 10% FBS, 0.5% ITS‐G, and 40 ng/mL Dexamethasone, along with 1% penicillin–streptomycin (P/S) and purchased from Procell (Catalog number: CM‐0602, Wuhan, China). In DMEM medium, TCMK1 cells were maintained with 10% FBS and 1% P/S.

### 2.7 Cell Viability Assay

Log‐phase cells were collected, diluted to a concentration of 5 × 10^4^ cells/mL, and seeded at 100 μL per well into a 96‐well plate, followed by overnight incubation. After the cells adhered, CMP solutions of varying concentrations were added to the experimental group. After 48 h of culture, a volume of 10 μL of CCK‐8 solution (KGA9310, KeyGEN BioTECH) was introduced into each well, followed by incubation of the cells for 1 to 2 h. Subsequently, the absorbance at 450 nm was measured using a microplate reader, and cell viability was subsequently calculated.

### 2.8 Apoptosis Assay

2 × 10^5^ logarithmic growth phase cells were seeded into a 6‐well culture plate. After an overnight culture, the cells were first treated with medium containing 100 mg/mL CMP for 12 h, followed by a 24‐h exposure to cisplatin. The PE‐Annexin V apoptosis assay kit (559763, BD) was used to assess the level of apoptosis, following the detection protocol outlined in the kit manual.

### 2.9 Assessment of IP3 and DAG Levels

The levels of inositol triphosphate (IP3) and diacylglycerol (DAG) were measured using competitive ELISA kits obtained from BBI Life Sciences Corporation (Cat: D751026) and Cell Biolabs Inc. (Cat: MET‐5028), respectively. After trypsin digestion, cells were harvested. A total of 2 × 10^6^ cells were lysed in 100 μL of PBS supplemented with protease inhibitors subjecting them to repeated freeze–thaw cycles. After centrifugation at 1500 *g* for 10 min, the supernatant was collected for analysis. After completing the sample incubation, reaction, and color development according to the provided instructions, the results were recorded at 450 nm using a microplate reader.

### 2.10 PKC and PLC Activity Assay

PKC and PLC activities were measured using the EnzChek Direct Phospholipase C Assay Kit (E10215, Invitrogen) and the PKC Assay Kit (ab139437, Abcam), respectively. Following the provided instructions, tissues and cells were homogenized and centrifuged, and the collected supernatant was used to detect PKC and PLC activities. After adjusting for protein levels determined by the BCA assay kit, the PKC and PLC enzyme activities were calculated.

### 2.11 IF Staining

Cells were seeded onto glass‐bottomed cell culture dishes and cultured until approximately 70% confluence. Immunofluorescence staining was performed after the cells were treated with CMP for 12 h. The cells underwent standard procedures as previously described (Zhang, Du, Shi, et al. 2022), which included fixation with 4% paraformaldehyde, membrane permeabilization with 0.2% Triton X‐100, blocking with 5% goat serum, incubation with the primary antibody (overnight, 4°C), and incubation with the secondary antibody (1 h, room temperature). Finally, after washing with PBS and covering an anti‐fluorescence decay blocking agent, images of the cells were obtained using fluorescence microscopy.

### 2.12 ROS and MMP Assay

Cells were seeded onto a 12‐well plate. After treatment with cisplatin and CMP, DCFH‐DA (S0033, Beyotime) and JC‐1 (C2006, Beyotime) dyes were used to detect cellular ROS and mitochondrial membrane potential (MMP) levels. Cells were then incubated with the working solution (DCFH‐DA reagent diluted 1000 times) at 37°C in the dark for 20 min, washed three times with PBS buffer, and subsequently photographed using a fluorescence microscope. For MMP detection, a 1 × JC‐1 staining working solution was prepared using a serum‐free medium, ensuring that the cells were fully covered with an adequate amount of the working solution. The cells were then incubated at 37°C in the dark for 20 min. Images were captured using a fluorescence microscope after washing with staining buffer. ImageJ software was used to analyze the levels of ROS and MMP in the cells.

### 2.13 siRNA Transfection and qPCR

Nrf2 siRNA was synthesized by Shanghai Sangon, and the sequence is presented in Table S1. The transfection steps were carried out based on previous experience (Zhang, Du, Xu, et al. 2022).

Tissues and cells were homogenized and lysed in Trizol solution, and total RNA was extracted using standard methods. After detecting the concentration and purity of the RNA, it was reverse‐transcribed into cDNA using the kit (AG0305, Shandong Sparkjade Biotechnology Co. Ltd.). The preparation of the reaction system and the setting of the reaction procedure were performed according to the qPCR kit (AH0101, Shandong Sparkjade Biotechnology Co. Ltd.) instructions, with results documented using a CF96 real‐time PCR instrument (Bio‐Rad). Gene expression analysis was performed using the 2^−ΔΔ*Ct*^ calculation method. The primers were synthesized by Sangon (Shanghai, China), and their sequences are provided in Table S2.

### 2.14 Nrf2‐ARE Reporter Gene Assay

Cells were cultured in all‐white 96‐well plates until they reached approximately 50% confluence. Subsequently, each well received 0.1 μg of the pARE firefly luciferase plasmid (D2112, Beyotime) and 0.01 μg of the pRL‐TK Renilla luciferase plasmid (D2760, Beyotime), which were co‐transfected into the cells utilizing Lipofectamine 3000 (L3000150, Thermo, USA). Forty‐eight hours following transfection, the cells were exposed to varying concentrations of CMP. Twelve hours post‐treatment, luciferase activity was measured using a reporter gene assay kit (KGE3308‐10, KeyGEN BioTECH), as previously reported (Zhang, Chen, et al. 2025).

### 2.15 Western Blotting

Cells and tissues were lysed and homogenized in lysis reagent (KGB5303, KeyGEN BioTECH). Following centrifugation, the supernatant was carefully collected, and its protein concentration was subsequently determined utilizing a BCA protein quantification assay kit (KGB2101, KeyGEN BioTECH). Protein supernatants were adjusted to 2 μg/μL with loading buffer (KGC4501, KeyGEN BioTECH) and heated in boiling water for 10 min. Equivalent amounts of protein were electrophoresed and then transferred to a PVDF membrane. Following the blocking procedure utilizing a 5% non‐fat milk solution in TBST, the membrane was incubated overnight at 4°C with the following primary antibodies (1:1000): α‐Tubulin (T6074, Sigma), Nrf2 (#12721, CST), p‐Nrf2 (PA5‐67520, Thermo Fisher), and Keap1 (#8047, CST). After washing with TBST buffer, the membrane was incubated with a secondary antibody (Beyotime, China) at room temperature for 1 h. The protein bands were visualized and imaged using a Bio‐Rad instrument, followed by incubation with ECL chemiluminescent substrate (WBKLS0500, Millipore). The relative protein levels were determined through grayscale analysis using ImageJ, with normalization against an internal reference protein.

### 2.16 Statistical Analysis

Statistical analysis and graphing were performed using GraphPad Prism 8 software. All experimental data are expressed as mean ± standard deviation (SD). Normality was assessed by the Shapiro–Wilk test, and homogeneity of variances was confirmed by Levene's test (all *p* > 0.05) prior to parametric analyses. For comparisons between two groups, an unpaired Student's *t*‐test was applied. For comparisons among three or more groups, one‐way analysis of variance (ANOVA) was used, followed by Tukey's post hoc test for all pairwise multiple comparisons. The significance level was set at *p* < 0.05.

## 3 Results

### 3.1 CMP Mitigate Cisplatin‐Induced Hepatorenal Injury

**FIGURE 1:**
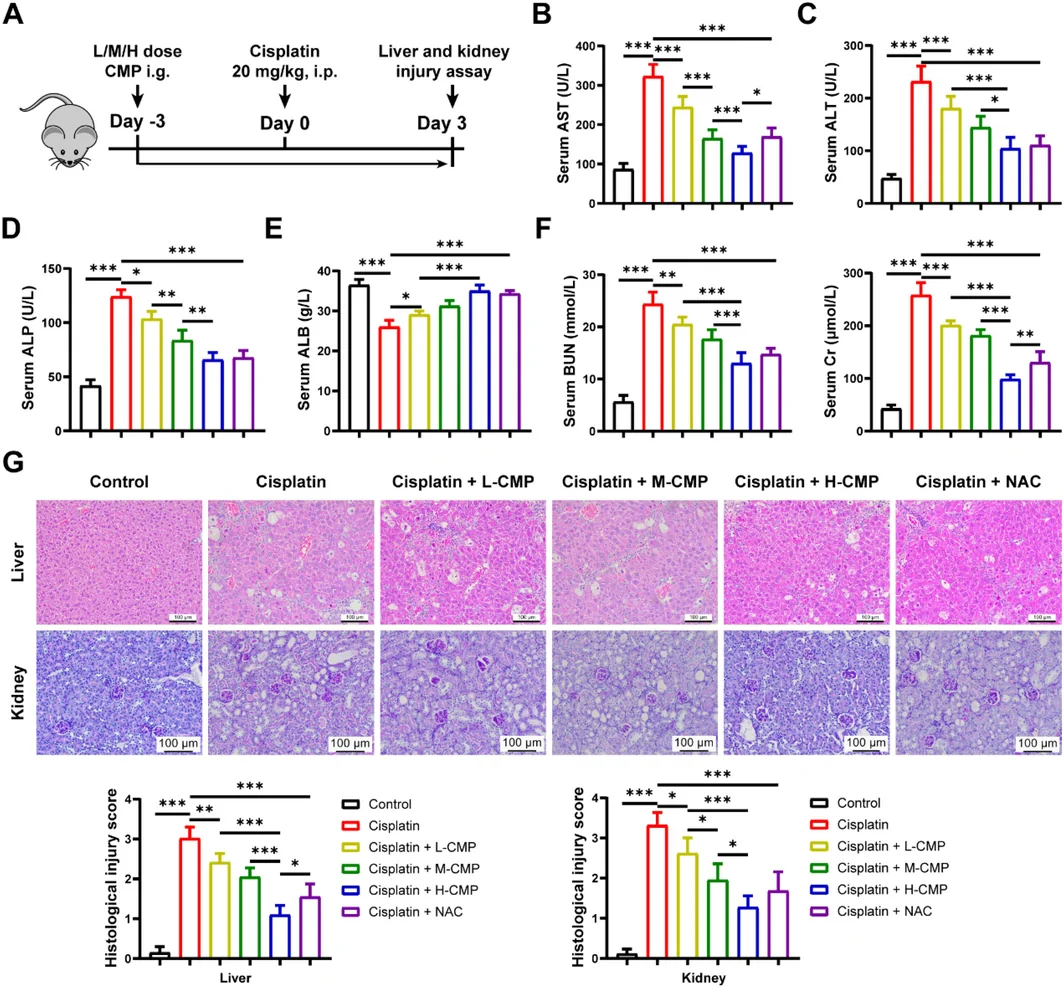
CMP mitigate cisplatin‐induced hepatorenal injury. (A) Schematic diagram of the modeling of cisplatin administration and CMP treatment. Detection of (B) ALT, (C) AST, and (D) ALP activity, as well as (E) ALB, (F) BUN, and Cr levels in serum. (G) HE staining and injury scoring of hepatorenal tissue. Scale bar: 100 μm. Data are presented as mean ± SD. *p < 0.05, **p < 0.01, ***p < 0.001.

As shown in Figure 1A, mice were administered cisplatin (i.p., 20 mg/kg) and subsequently treated with varying doses of CMP (low, medium, and high), with NAC serving as a positive control (Figure 1A). At the conclusion of the study, the hepatorenal functions of the mice were assessed by quantifying the levels of ALT, ALP, ALB, BUN, AST, and Cr in serum. The results showed that, the cisplatin‐treated group exhibited significantly elevated serum levels of ALT, AST, ALP, BUN, and Cr, while ALB levels were markedly reduced compared to the control group (Figure 1B–F). Treatment with CMP effectively mitigated the alterations in these biochemical markers induced by cisplatin, with a clear dose‐dependent relationship observed (Figure 1B–F). Furthermore, histological examination via HE staining of liver tissues revealed that cisplatin treatment resulted in indistinct hepatocyte boundaries and inflammatory cell infiltration surrounding blood vessels (Figure 1G). Similarly, HE staining of kidney tissues demonstrated that cisplatin led to the formation of distinct droplets within proximal tubular cells, necrosis of tubular epithelial cells, and interstitial edema (Figure 1G). In the CMP treatment group, the cisplatin‐induced damage to hepatorenal tissues was significantly ameliorated, with the high‐dose CMP exhibiting the most pronounced protective effect (Figure 1G). These findings suggest that CMP have the potential to attenuate cisplatin‐induced hepatorenal damage.

### 3.2 CMP Alleviate Cisplatin‐Induced Peripheral and Hepatorenal Inflammation

**FIGURE 2:**
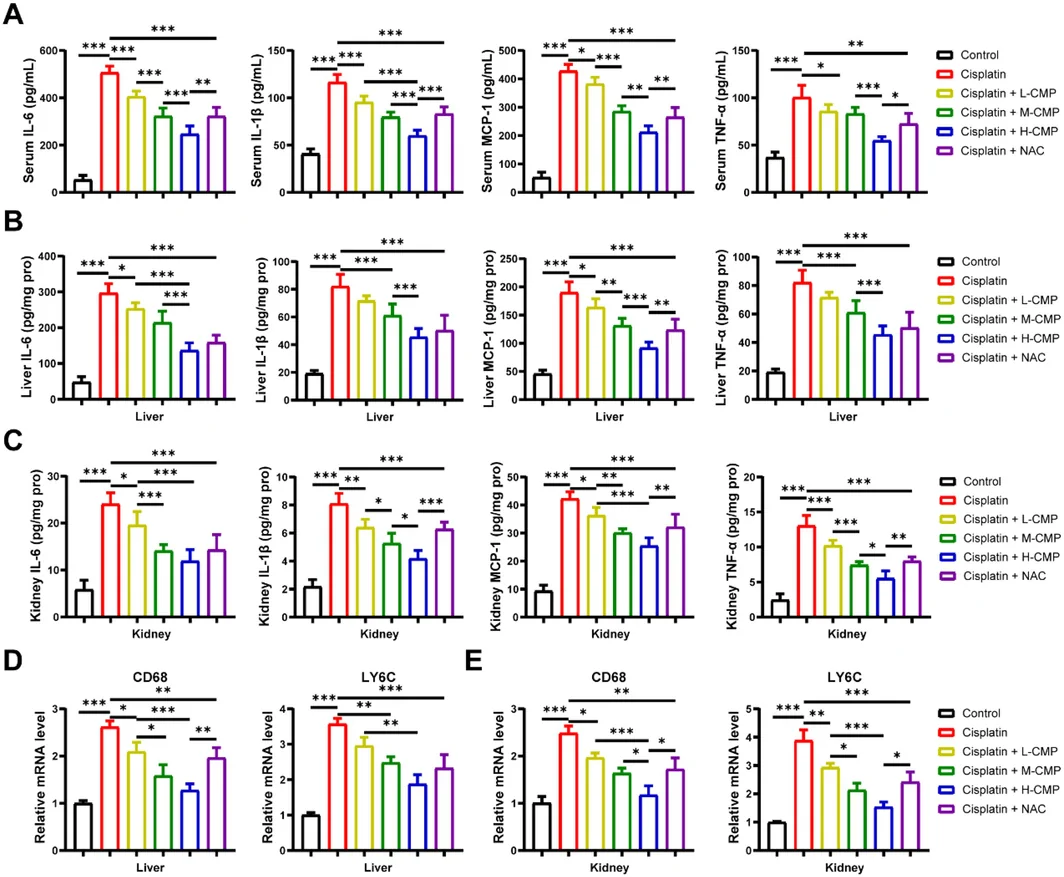
CMP alleviate cisplatin‐induced peripheral and hepatorenal inflammation. (A–C) Detection of IL‐1β, IL‐6, MCP‐1, and TNF‐α protein levels in serum and hepatorenal tissues. (D, E) Detection of CD68 and LY6C mRNA levels in hepatorenal tissues. Data are presented as mean ± SD. *p < 0.05, **p < 0.01, ***p < 0.001.

In the organ toxicity induced by cisplatin, the increase in inflammatory cytokines and the infiltration of inflammatory cells may result in secondary tissue damage (Santabarbara et al. 2016). Consequently, we examined the changes in inflammatory factor levels in peripheral and liver‐kidney tissues. As shown in Figure 2A–C, the group administered cisplatin demonstrated a marked elevation in inflammatory mediators, specifically IL‐6, IL‐1β, TNF‐α, and MCP‐1, in comparison to the control group. Notably, CMP treatment effectively attenuated the cisplatin‐induced elevation of these inflammatory factors in a dose‐dependent manner (Figure 2A–C). Additionally, the mRNA levels of CD68 and LY6C, which serve as markers for monocytes and macrophages, were assessed in hepatorenal tissues using quantitative PCR. The results revealed a significant upregulation of CD68 and LY6C mRNA levels in the hepatorenal tissues of the cisplatin group compared to the control group, whereas CMP treatment resulted in a substantial decrease in their expression (Figure 2D,E). These findings suggest that CMP mitigates the inflammatory response in peripheral and liver‐kidney tissues triggered by cisplatin, supporting the observed pathological outcomes.

### 3.3 CMP Mitigate Cisplatin‐Induced Hepatorenal Oxidative Damage

**FIGURE 3:**
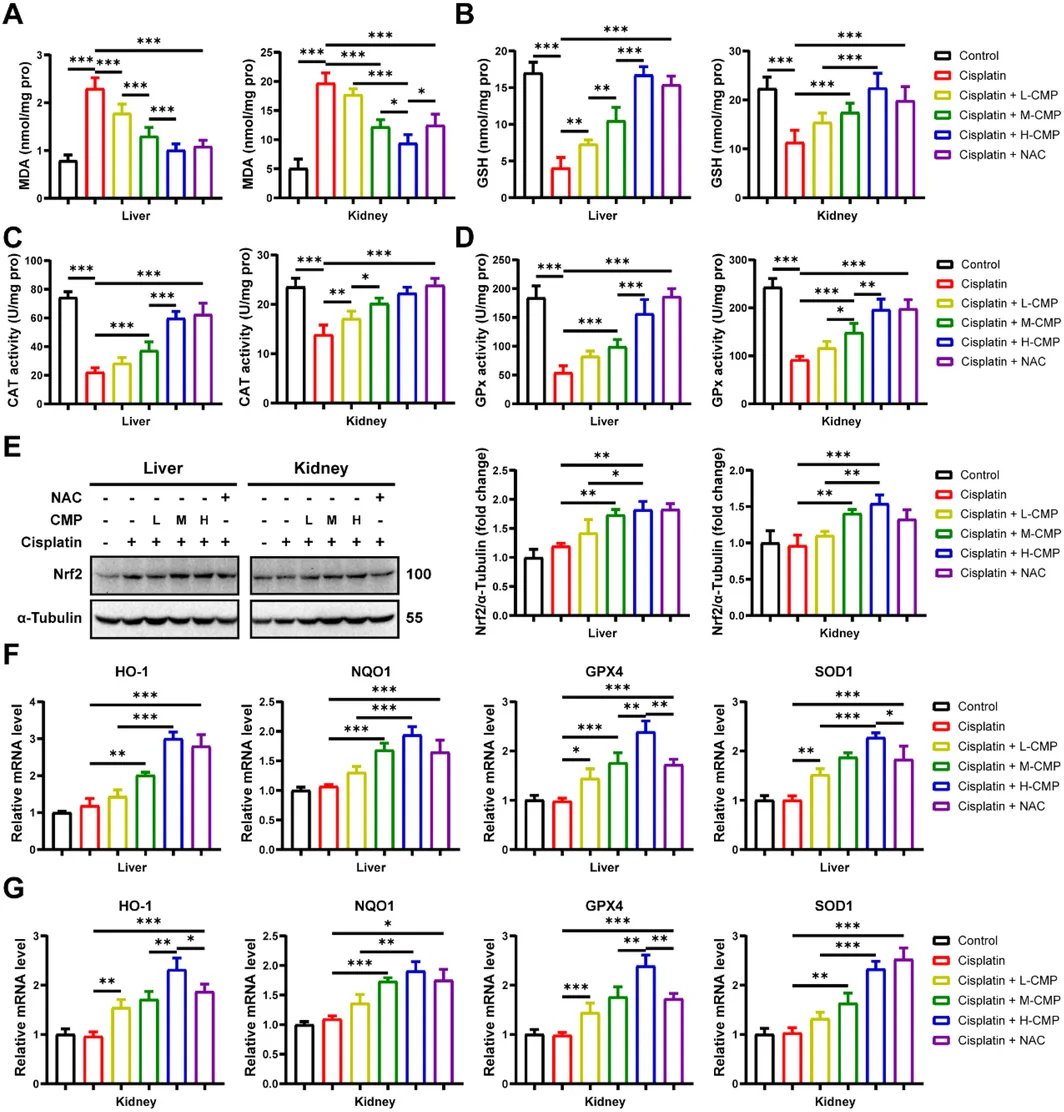
CMP mitigate cisplatin‐induced hepatorenal oxidative damage. Detection of (A) MDA, (B) GSH, (C) CAT, (D) GPx, and (E) Nrf2 protein levels in hepatorenal tissues. (F, G) Assessment of mRNA expression levels of NQO1, HO‐1, SOD1, and GPX4 in hepatorenal tissues. Data are presented as mean ± SD. *p < 0.05, **p < 0.01, ***p < 0.001.

In addition to inflammation, oxidative stress contributes to cellular damage caused by cisplatin (Agrawal and Jain 2025). Consequently, we evaluated the levels of the oxidative stress biomarker MDA in hepatorenal tissues. The findings demonstrated that CMP administration markedly decreased the cisplatin‐induced production of MDA (Figure 3A). Moreover, cisplatin not only diminished GSH levels in the hepatorenal tissues but also decreased the activities of CAT and GPx (Figure 3B–D). Treatment with CMP successfully reinstated both GSH levels, CAT and GPx activities (Figure 3B–D). Additionally, following CMP treatment, an elevation in Nrf2 protein levels was detected, which serves as a vital intracellular transcriptional regulator of antioxidant responses, in hepatorenal tissues (Figure 3E). Correspondingly, the expression levels of its downstream antioxidant genes, including NQO1, HO‐1, SOD1, and GPX4, were also significantly enhanced in these tissues (Figure 3F,G). These findings indicate that CMP mitigate oxidative damage caused by cisplatin in hepatorenal tissues.

### 3.4 CMP Mitigate Cisplatin‐Induced Oxidative Damage of Hepatorenal Cells In Vitro

**FIGURE 4:**
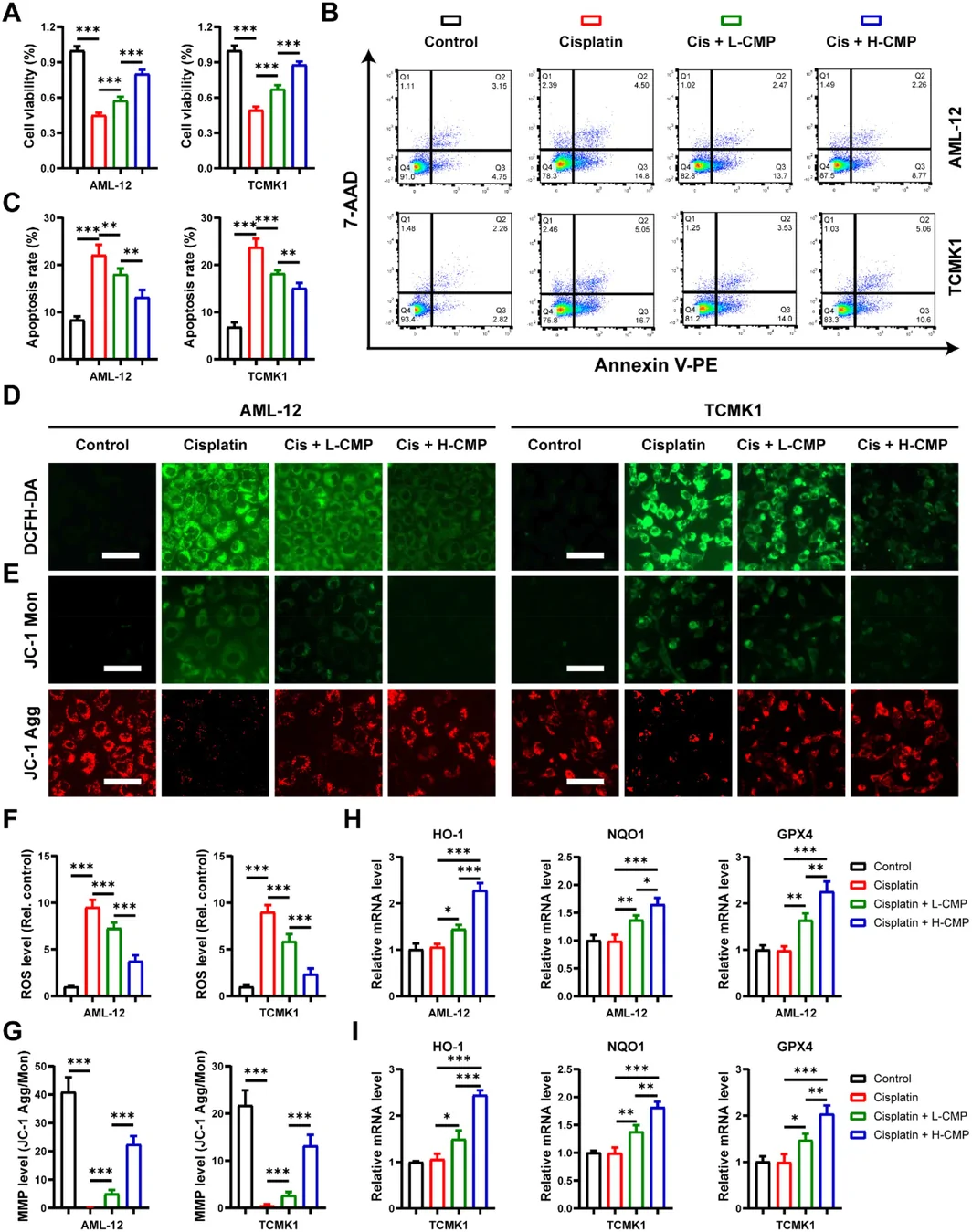
CMP mitigate cisplatin‐induced oxidative damage of hepatorenal cells in vitro. Detection of (A) cell viability, (B, C) apoptosis, (D, F) ROS, and (E, G) MMP levels in TCMK1 and AML‐12 cells. Scale bar: 100 μm. (H, I) Assessment of NQO1, HO‐1, and GPX4 mRNA levels in TCMK1 and AML‐12 cells. Data are presented as mean ± SD. *p < 0.05, **p < 0.01, ***p < 0.001.

We further validated the protective effects of CMP in vitro. First, we treated AML‐12 and TCMK1 cells with varying concentrations of CMP (0–100 μg/mL) and observed no significant alterations in cell viability (Figure S1A). Based on previous studies (Zhang, Chen, et al. 2025), we selected 50 μg/mL and 100 μg/mL as the low and high concentrations for subsequent CMP treatment experiments. Utilizing CCK‐8 assays to assess cell viability and Annexin V‐PE/7‐AAD for apoptosis detection, results revealed that CMP treatment significantly inhibited the decline in cell viability and the increase in apoptosis induced by cisplatin in both AML‐12 and TCMK1 cells, with the higher concentration of CMP exhibiting a more pronounced effect than the lower concentration (Figure 4A–C). Furthermore, we evaluated the levels of ROS and MMP using the fluorescent probes DCFH‐DA and JC‐1. The results demonstrated that cisplatin treatment significantly elevated ROS production and diminished MMP levels, whereas CMP treatment effectively reversed these alterations (Figure 4D–G). The measurement of the lipid oxidation marker MDA and the antioxidant marker GSH demonstrated that CMP significantly enhanced the antioxidant capacity of AML‐12 and TCMK1 cells while concurrently inhibiting oxidative stress induced by cisplatin (Figure S1B,C). Moreover, quantitative PCR analysis of the expression of antioxidant genes regulated by Nrf2 showed that CMP significantly raised the transcriptional levels of the antioxidant genes NQO1, HO‐1, and GPX4 (Figure 4H,I). Collectively, these findings suggest that CMP exerts a protective effect against oxidative damage in hepatorenal cells induced by cisplatin.

### 3.5 Nrf2 Mediates CMP Resistance to Cisplatin‐Induced Hepatorenal Cells Damage

**FIGURE 5:**
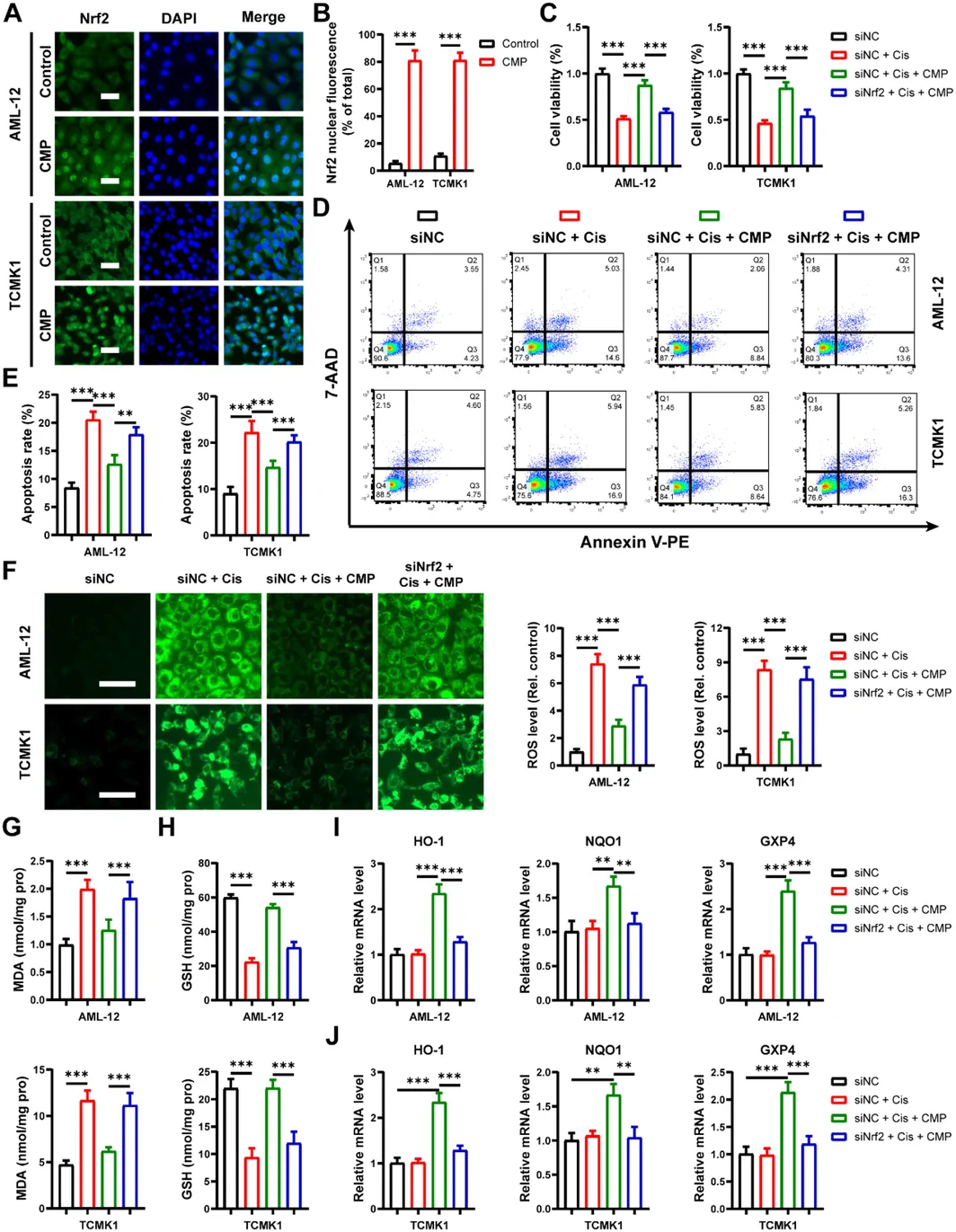
Nrf2 mediates CMP resistance to cisplatin‐induced hepatorenal cell damage. (A, B) IF staining of Nrf2 in TCMK1 and AML‐12 cells. Scale bar: 50 μm. Detection of (C) cell viability, (D, E) apoptosis, (F) ROS, (G) MDA, and (H) GSH levels in TCMK1 and AML‐12 cells. Scale bar: 100 μm. (I, J) Detection of NQO1, HO‐1, and GPX4 mRNA levels. Data are presented as mean ± SD. **p < 0.01, ***p < 0.001.

In vivo studies revealed that CMP have the capacity to enhance the levels of Nrf2 protein and increase the levels of downstream regulatory antioxidant genes. Consequently, we hypothesize that CMP may activate Nrf2. The findings from Nrf2 immunofluorescence staining conducted in vitro demonstrated that CMP markedly facilitates the nuclear translocation of Nrf2 (Figure 5A,B). Additionally, the protective effects of CMP against cisplatin‐induced reductions in cell viability and apoptosis were blocked following siRNA‐mediated interference with Nrf2 expression (Figure 5C–E, Figure S2). Similarly, the knockdown of Nrf2 diminished the ability of CMP to inhibit the production of ROS and MDA induced by cisplatin, as well as its ability to enhance GSH levels (Figure 5F–H). Quantitative PCR results further indicated that the silencing of Nrf2 inhibited the transcriptional expression of NQO1, HO‐1, and GPX4 activated by CMP (Figure 5I,J). These findings suggest that Nrf2 is involved in the protective effects of CMP against cisplatin‐induced oxidative damage in hepatorenal cells.

### 3.6 CMP Induce Nrf2 Phosphorylation Activation by Activating the PLC‐PKC Signaling Pathway

**FIGURE 6:**
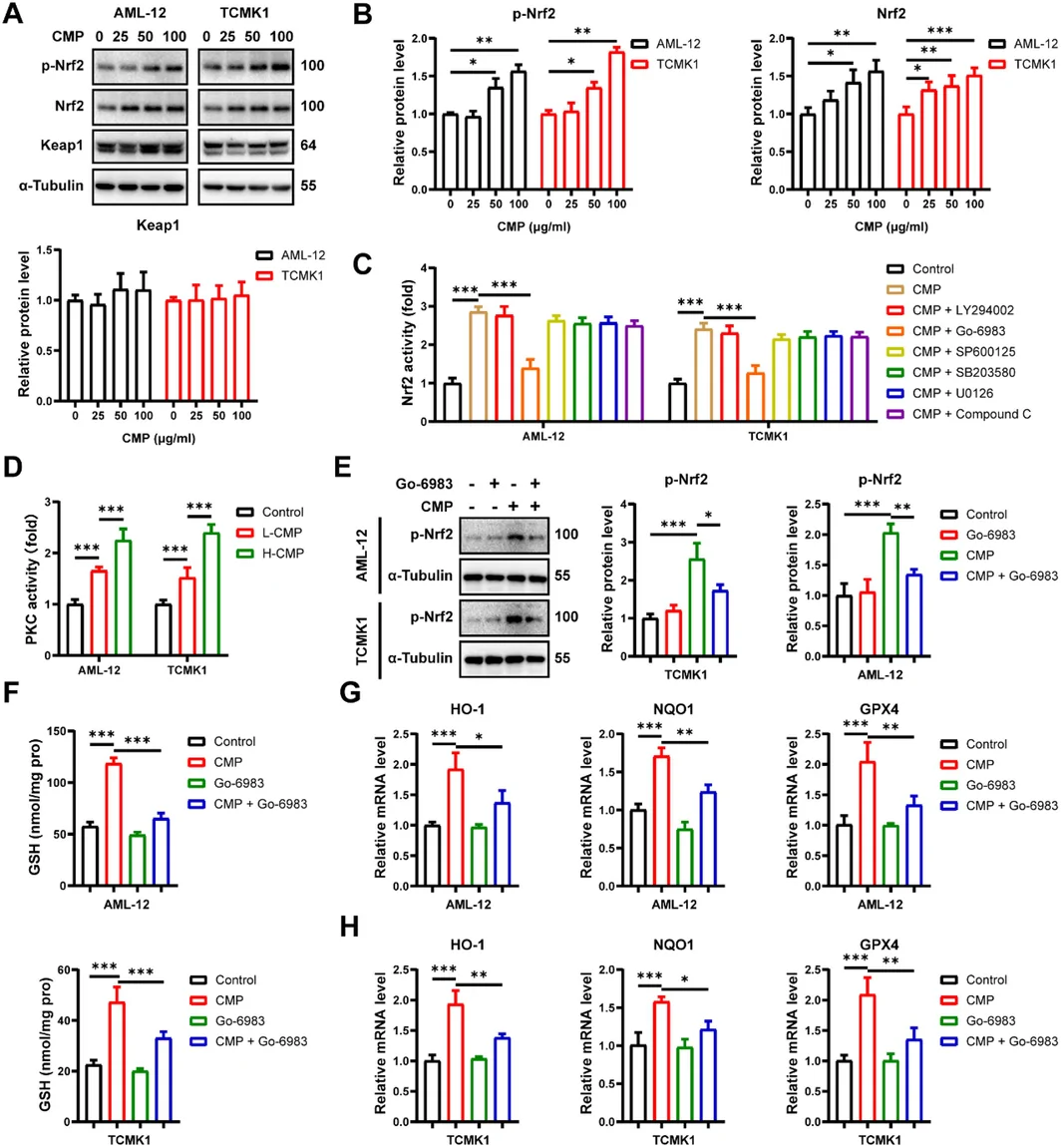
PKC mediates CMP‐induced Nrf2 activation in hepatorenal cells. (A, B) Detection of Keap‐1, p‐Nrf2, and Nrf2 protein levels in AML‐12 and TCMK1 cells. (C, D) Detection of Nrf2 and PKC activities. (E, F) Assessment of p‐Nrf2 protein and GSH levels in AML‐12 and TCMK1 cells. (G, H) Assessment of mRNA expression levels of HO‐1, GPX4, and NQO1 was conducted in AML‐12 and TCMK1 cells. Data are presented as mean ± SD. *p < 0.05, **p < 0.01, ***p < 0.001.

To elucidate the mechanism by which CMP facilitates the activation of Nrf2, we conducted an analysis of the protein levels of Nrf2, p‐Nrf2, and Keap1 in AML‐12 and TCMK1 cells following CMP treatment. CMP significantly elevated the levels of p‐Nrf2 and Nrf2, while the level of Keap1 remained unchanged (Figure 6A). This observation, in conjunction with a review of existing literature (Vomund et al. 2017), suggests that CMP may enhance the transcriptional activity of Nrf2 by promoting its phosphorylation. Furthermore, treatment with inhibitors targeting upstream kinases revealed that the inhibition of PKC activity significantly impeded the CMP‐induced enhancement of Nrf2 activity (Figure 6B). Additionally, assessment of PKC enzyme activity demonstrated that CMP treatment significantly increased PKC activity in both AML‐12 and TCMK1 cells (Figure 6C). Following pre‐treatment with the PKC inhibitor Go‐6983, we observed a substantial decrease in the CMP‐induced elevation of p‐Nrf2 protein levels and GSH production in these cell lines (Figure 6D,E). Moreover, Go‐6983 also inhibited CMP‐induced transcriptional expression of NQO1, HO‐1, and GPX4 (Figure 6F,G).

**FIGURE 7:**
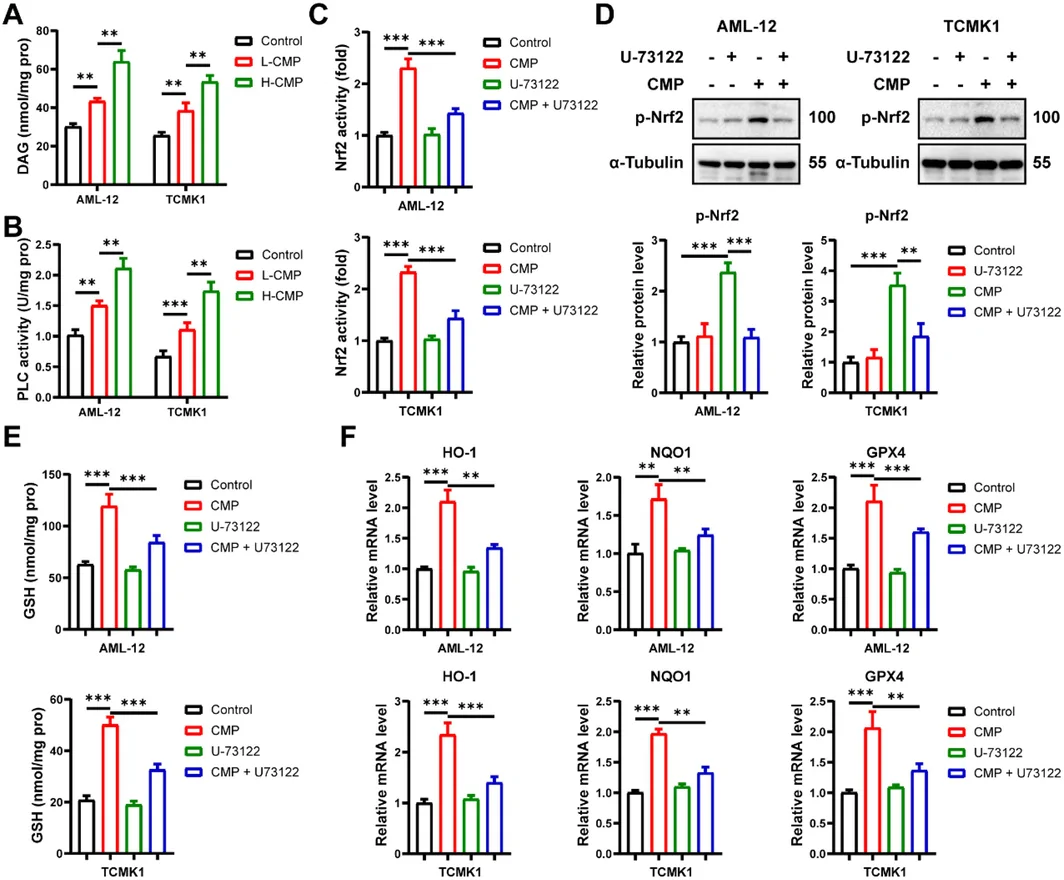
CMP induces the activation of PKC through the PLC pathway. (A, B) Detection of DAG levels and PLC activity in AML‐12 and TCMK1 cells. Assessment of (C) Nrf2 activity, (D) p‐Nrf2 protein levels, and (E) GSH levels. (F) An evaluation of the mRNA expression levels of NQO1, HO‐1, and GPX4 in AML‐12 and TCMK1 cells. Data are presented as mean ± SD. **p < 0.01, ***p < 0.001.

Numerous studies have demonstrated that PKC is activated through the synergistic effects of DAG and calcium ions (Steinberg 2015). DAG, a second messenger, is produced via the enzymatic hydrolysis of phosphatidylinositol 4,5‐bisphosphate (PIP2) catalyzed by PLC (Steinberg 2015). Consequently, we assessed the levels of DAG and the activity of the PLC enzyme in AML‐12 and TCMK1 cells following treatment with CMP. The results showed that CMP significantly enhanced intracellular DAG levels and PLC enzyme activity (Figure 7A,B). Furthermore, the application of U‐73112, a PLC enzyme activity inhibitor, resulted in a marked reduction of CMP‐induced elevation in Nrf2 transcriptional activity and p‐Nrf2 protein levels (Figure 7C,D). Similarly, this inhibition also obstructed the CMP‐induced increase in GSH levels and the transcriptional levels of antioxidant genes (GPC4, HO‐1, and NQO1) (Figure 7E,F). These results collectively suggest that CMP induces the phosphorylation and activation of Nrf2 via the PLC‐PKC signaling pathway.

### 3.7 Inhibition of Nrf2 Blocks the Protective Effects of CMP Against Cisplatin‐Induced Hepatorenal Damage In Vivo

**FIGURE 8:**
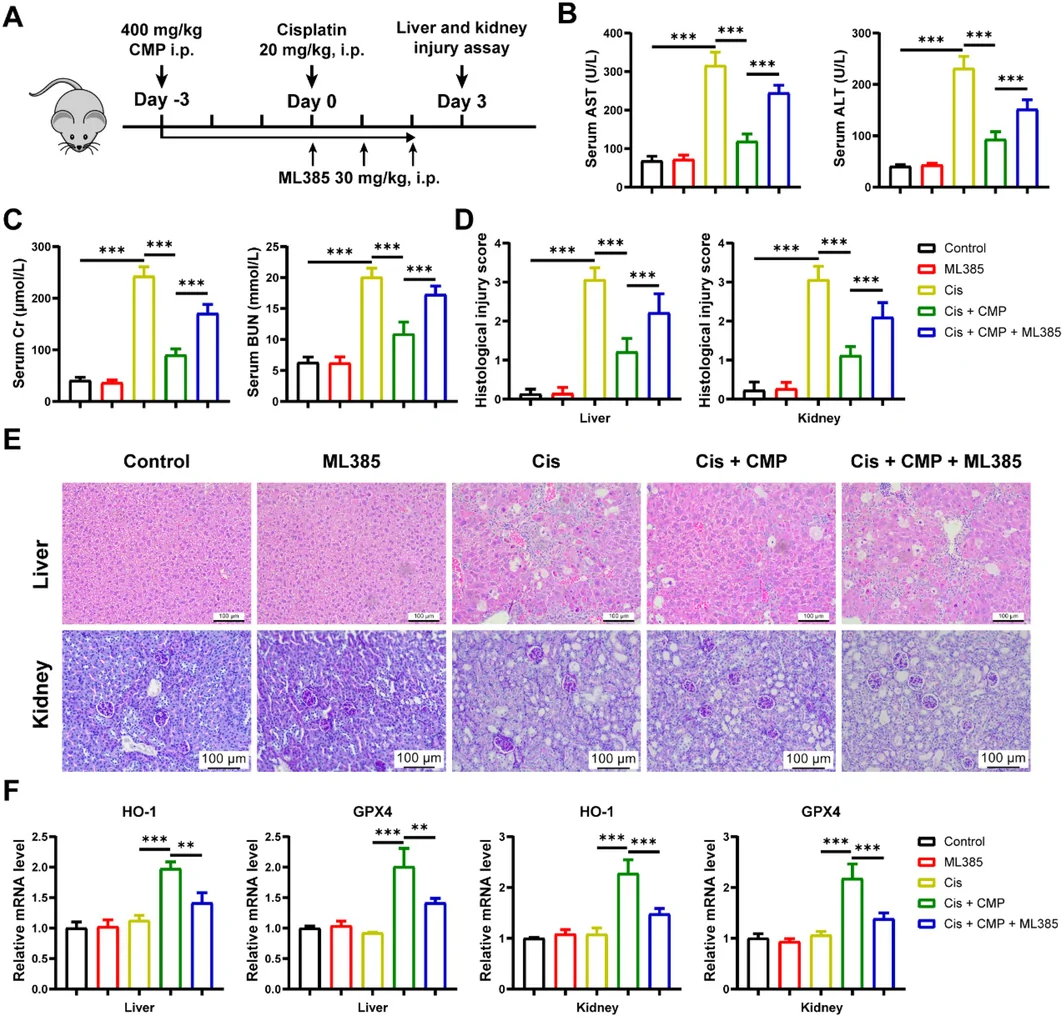
CMP mitigates cisplatin‐induced hepatorenal injury dependent on Nrf2 in vivo. (A) Schematic diagram illustrating the administration of cisplatin, CMP, and ML385. (B, C) Detection of AST, ALT, Cr, and BUN levels in serum. (D, E) HE staining and damage scoring of hepatorenal tissue. Scale bar: 100 μm. (F) Evaluation of the mRNA expression levels of HO‐1 and GPX4 in hepatorenal tissues. Data are presented as mean ± SD. **p < 0.01, ***p < 0.001.

To evaluate the dependence of CMP's hepatoprotective and nephroprotective effects on Nrf2, an Nrf2 inhibitor was utilized in vivo experiments. Prior to the administration of CMP, the mice received an intraperitoneal injection of ML385 (Figure 8A). The results indicated that the liver and kidney functions of the mice that were solely administered ML385 remained unchanged, suggesting the safety of the ML385 injection (Figure 8B,C). However, ML385 significantly diminished the protective effects of CMP against cisplatin‐induced hepatic and renal injury, as evidenced by a reduced efficacy of CMP in mitigating the elevated levels of AST, ALT, BUN, and Cr (Figure 8B,C). Histological examinations further revealed that following pretreatment with ML385, CMP were unable to effectively prevent damage to liver and kidney tissues caused by cisplatin (Figure 8D,E). Additionally, quantitative PCR analyses demonstrated that ML385 inhibited HO‐1 and GPX4 expression in liver and kidney tissues stimulated by CMP (Figure 8F). Consequently, these results provide in vivo evidence that the protective mechanism of CMP against cisplatin‐induced liver and kidney damage is dependent on the Nrf2 signaling pathway.

## 4 Discussion

The side effects of chemotherapy present an unavoidable challenge in cancer treatment. However, the blind use of additional medications to alleviate these side effects inevitably leads to other potential side effects, such as headaches and constipation caused by anti‐nausea drugs (Society of Urology of Zhejiang Medical Association and Committee of Breast Cancer of Zhejiang Anticancer Association 2022), bone pain resulting from white blood cell‐boosting medications (Agrawal and Jain 2025), and the impact of hepatorenal antioxidant protectants on tumor treatment (Wieland et al. 2021). Polysaccharide drugs generally exhibit good biocompatibility and low toxicity (Hou et al. 2026; Wan et al. 2022; Yang et al. 2026). Several polysaccharide drugs are already used clinically in China for anti‐tumor purposes, enhancing the body's immunity and mitigating the side effects of radiotherapy and chemotherapy (Shui et al. 2025; Wan et al. 2022). These include ginseng polysaccharides, lentinan, astragalus polysaccharides, *Tremella fuciformis* polysaccharides, *Polyporus umbellatus* polysaccharide, and *Ganoderma lucidum* polysaccharide (Mohammed et al. 2021; Shui et al. 2025; Wan et al. 2022).

PCP has exhibited protective properties in multiple murine models of renal and hepatic pathologies. It mitigates both alcohol‐induced and non‐alcoholic steatohepatitis by modulating gut microbiota, preserving the integrity of the intestinal barrier, attenuating oxidative stress, and reducing endotoxin translocation (Jiang et al. 2022; Tan et al. 2022; Ye et al. 2022). PCP has exhibited protective effects against CCl4 and acetaminophen induced liver damage in mice (Cheng et al. 2021; Wu et al. 2018, 2019). Additionally, PCP has shown nephroprotective properties in hyperuricemic mice (Liang et al. 2021), as it decreased levels of pro‐inflammatory cytokines and enhanced renal morphology in models of adenine‐induced chronic kidney disease (CKD) (Fu et al. 2021; Zhao et al. 2013). Our findings indicate that CMP can effectively inhibit cisplatin‐induced hepatorenal damage in mice, reducing the expression of inflammatory factors and immune cell infiltration in peripheral and hepatorenal tissues. Additionally, CMP can significantly enhance the antioxidant capacity of hepatorenal tissues, thereby alleviating cisplatin‐induced oxidative damage.

Nrf2 serves as the principal signaling pathway for the regulation of oxidative stress and the inflammatory response, playing a vital role in the management of cellular oxidative stress and the maintenance of tissue homeostasis in the context of damage (Zhang 2025). Key antioxidant genes associated with this pathway include HO‐1, SOD, GPX4, and NQO1 (Vomund et al. 2017). Nrf2 knockout mice demonstrate significantly pronounced adverse effects resulting from chemotherapy agents, which encompass renal impairment, hepatic injury, neurotoxicity, and ototoxicity (Fan et al. 2020; Ma et al. 2021; Sagrac et al. 2024; Wang et al. 2022; Zhang, Hu, et al. 2025). A multitude of studies has indicated that various Nrf2 activators confer protective effects on hepatic and renal tissues against cisplatin‐induced damage (Mirzaei et al. 2021). For instance, both natural and synthetic compounds, such as curcumin (Jin et al. 2025), resveratrol (Hao et al. 2016), and N‐acetylcysteine (Li et al. 2019; Zavala‐Valencia et al. 2024), exerting antioxidant, anti‐apoptotic, and anti‐inflammatory effects that mitigate the toxicity of cisplatin on the liver and kidneys by activating the Nrf2 pathway (Wang et al. 2024). Our findings demonstrate that CMP can effectively diminish oxidative stress, inflammatory responses, and cellular apoptosis induced by cisplatin through the activation of the Nrf2 signaling pathway, thereby protecting hepatorenal tissues from injury.

Nrf2 activation primarily occurs via two distinct pathways: the Keap1‐dependent pathway and the Keap1‐independent pathway (Vomund et al. 2017). In the Keap1‐dependent pathway, exposure of cells to oxidative or electrophilic stress results in modifications to the cysteine residues on Keap1, which subsequently leads to a conformational change that facilitates the release of Nrf2 (Zhang 2025). Conversely, in the Keap1‐independent pathway, upstream kinases phosphorylate Nrf2, which promotes its dissociation from Keap1 and enhances its transcriptional activity (Vomund et al. 2017). Previous studies have indicated that polysaccharides derived from *Poria cocos* can activate Nrf2 signaling (Chen et al. 2024; Zhao et al. 2020; Zhou et al. 2024); however, the precise mechanism of activation remains unclear. The findings of the present study demonstrate that CMP induce Nrf2 activation through a Keap1‐independent mechanism. Specifically, CMP activate the PLC‐PKC signaling pathway, which results in the phosphorylation and activation of Nrf2, thereby promoting the expression of its downstream antioxidant genes.

Unlike canonical Keap1‐dependent Nrf2 activation, the PLC‐PKC‐mediated Nrf2 phosphorylation observed here enables rapid and reversible activation. This kinetic feature may avoid prolonged Nrf2 stabilization, thereby potentially reducing the risk of chemoresistance and unintended tumor protection associated with constitutive Nrf2 activation. The current clinical standard for nephroprotection against cisplatin toxicity involves aggressive hydration combined with forced diuresis (Elmorsy et al. 2024). However, this strategy is frequently contraindicated in patients with pre‐existing heart failure, advanced age, or impaired renal function, due to the significant risks posed by volume overload (Sikking et al. 2024). In such populations, oral administration of CMP may offer a mechanism‐based alternative that does not rely on volume expansion. Nevertheless, direct comparative clinical trials between CMP and hydration protocols are necessary to define its therapeutic role.

Several limitations exist in the present study. First, all animal experiments were performed in non‐tumor‐bearing mice, leaving unclear whether CMP interferes with cisplatin's antitumor activity; subsequent verification using syngeneic tumor or PDX models is required to monitor tumor growth and survival upon combination therapy. Second, direct comparisons between CMP and raw PCP were absent. Despite documented improved solubility and bioavailability via carboxymethylation, equivalent hepatorenal protection from PCP cannot be ruled out, necessitating side‐by‐side assays. Third, the pan‐PKC inhibitor Go‐6983 failed to pinpoint the exact PKC subtype governing Nrf2 activation, and isoform‐specific knockout or siRNA tools are needed to distinguish functional PKC isoforms such as PKCδ. Lastly, only short‐term protective effects of CMP were characterized. Further long‐term assays involving multiple cisplatin dosing cycles and pharmacokinetic analyses for CMP‐cisplatin drug interactions are indispensable.

## 5 Conclusion

Our results demonstrated that CMP enhanced the antioxidant and anti‐inflammatory capacities of the hepatorenal system, providing protection against cisplatin‐induced damage. Furthermore, we identified Nrf2 activation as a critical mechanism underlying its protective effects, with PLC‐PKC serving as the upstream signaling pathway. These findings indicate that CMP could function as a supplementary treatment alongside cisplatin chemotherapy to mitigate liver and kidney toxicity.

## Author Contributions

**Liang He:** investigation, visualization, formal analysis, funding acquisition, writing – original draft. **Shuo Chen:** investigation, visualization. **Baoming Wu:** funding acquisition. **Jiong Gu:** investigation, visualization. **Conghan Li:** investigation. **Xianfan Du:** investigation. **Jun Chu:** project administration, conceptualization, writing – review and editing. **Min Yang:** conceptualization, project administration, writing – review and editing. **Hui Hou:** writing – review and editing, funding acquisition, conceptualization.

## Funding

This work was supported by Research Fund of Anhui Institute of Translational Medicine (2023zhyx‐C84), Anhui Clinical and Translational Medicine Research Project (202204295107020030), National Natural Science Foundation of China (82570695).

## Conflicts of Interest

The authors declare no conflicts of interest.

## Supporting information


**Table S1:** Sequences of small interfering RNA.
**Table S2:** Sequences of primer.
**Figure S1:** (A) AML‐12 and TCMK1 cells were treated with different concentrations of CMP for 48 h, and then cell viability was assessed using the CCK‐8 assay. (B, C) AML‐12 and TCMK1 cells were treated with low concentration (50 μg/mL) and high concentration (100 μg/mL) CMP for 48 h, and then the levels of MDA and GSH in the cells were measured. **p < 0.01, ***p < 0.001.
**Figure S2:** AML‐12 and TCMK1 cells were transfected with Nrf2 siRNA for 24 h, followed by the quantification of Nrf2 mRNA expression levels using qPCR. ***p < 0.001.

## Data Availability

The data that support the findings of this study are available from the corresponding author upon reasonable request.
