# Supplementary material for: Carboxymethylated Poria cocos Polysaccharides Mitigate Cisplatin‐Induced Hepatorenal Injury by Activating the PLC‐PKC‐Nrf2 Signaling Pathway
Source: Food Sci Nutr. 2026 Sep 5;14(9):e72342. doi: 10.1002/fsn3.72342 (PMC13545543; doi:10.1002/fsn3.72342)
Supplement: Supplementary file 1 [file FSN3-14-e72342-s001.docx]

Supplementary Materials for

**Carboxymethylated *Poria cocos* polysaccharides** **mitigate cisplatin-induced** **hepatorenal injury by activating the PLC-PKC-Nrf2 signaling pathway**

Liang He ^a, 1^, Shuo Chen ^b, 1^, Baoming Wu ^c^, Jiong Gu ^a^, Conghan Li ^a^, Xianfan Du ^a^, Jun Chu ^b,^ *, Min Yang ^d,^ *, Hui Hou ^a,^ *

^a^ Department of General Surgery, The Second Affiliated Hospital of Anhui Medical University, Hefei 230601, Anhui, China.

^b^ Key Laboratory of Xin'an Medicine, Ministry of Education, Anhui University of Chinese Medicine, Hefei 230038, Anhui, China

^c^ Inflammation and Immune Mediated Diseases Laboratory of Anhui Province, Anhui Medical University, Hefei 230032, Anhui, China

^d^ The 2nd Department of Intensive Care Unit, The Second Affiliated Hospital of Anhui Medical University, Hefei 230601, China

^1^ Liang He and Shuo Chen contributed equally to this work.

* Corresponding authors.

E-mail addresses: chuj@ahtcm.edu.cn (J. Chu), yangmin@ahmu.edu.cn (M. Yang), efy101303@fy.ahmu.edu.cn (H. Hou).

**Supplementary Tables**

**Table S1. Sequences of small interfering RNA**

| Target gene | Sequences |
| --- | --- |
| Mouse Nrf2 | 5’-UGGCAACUCCAAGUCCAUC-3’ |

**Table S2. Sequences of primer.**

| Target gene | Sequences |
| --- | --- |
| Mouse TNF-α | F: 5’-CAGGCGGTGCCTATGTCTC-3’  R: 5’-CGATCACCCCGAAGTTCAGTAG-3’ |
| Mouse IL-1β | F: 5’-GAAATGCCACCTTTTGACAGTG-3’  R: 5’-TGGATGCTCTCATCAGGACAG-3’ |
| Mouse IL-6 | F 5’-TCTATACCACTTCACAAGTCGGA-3’ |
|  | R 5’-GAATTGCCATTGCACAACTCTTT-3’ |
| Mouse MCP-1 | F 5’-TTAAAAACCTGGATCGGAACCAA-3’ |
|  | R 5’-GCATTAGCTTCAGATTTACGGGT-3’ |
| Mouse HO-1 | F 5’-GATAGAGCGCAACAAGCAGAA-3’ |
|  | R 5’-CAGTGAGGCCCATACCAGAAG-3’ |
| Mouse NQO1 | F 5’-AGGATGGGAGGTACTCGAATC-3’ |
|  | R 5’-TGCTAGAGATGACTCGGAAGG-3’ |
| Mouse GPX4 | F 5’-AGTACAGGGGTTTCGTGTGC-3’ |
|  | R 5’-CATGCAGATCGACTAGCTGAG-3’ |
| Mouse SOD1 | F 5’-AACCAGTTGTGTTGTCAGGAC-3’ |
|  | R 5’-CCACCATGTTTCTTAGAGTGAGG-3’ |
| Mouse CD68 | F 5’-TGTCTGATCTTGCTAGGACCG-3’ |
|  | R 5’-GAGAGTAACGGCCTTTTTGTGA-3’ |
| Mouse LY6C | F 5’-GCAGTGCTACGAGTGCTATGG-3’ |
|  | R 5’-ACTGACGGGTCTTTAGTTTCCTT-3’ |
| Mouse β-actin | F 5’-GACCTCTATGCCAACACAGT-3’ |
|  | R 5’-AGTACTTGCGCTCAGGAGGA-3’ |
| Mouse Nrf2 | F 5’-TCTTGGAGTAAGTCGAGAAGTGT-3’ |
|  | R 5’-GTTGAAACTGAGCGAAAAAGGC-3’ |

**Supplementary Figures**

**Figure. S1** (A) AML-12 and TCMK1 cells were treated with different concentrations of CMP for 48 hours, and then cell viability was assessed using the CCK-8 assay. (B-C) AML-12 and TCMK1 cells were treated with low concentration (50 μg/ml) and high concentration (100 μg/ml) CMP for 48 hours, and then the levels of MDA and GSH in the cells were measured. ***P* < 0.01, ****P* < 0.001.

**Figure. S2** AML-12 and TCMK1 cells were transfected with Nrf2 siRNA for 24 hours, followed by the quantification of Nrf2 mRNA expression levels using qPCR. ****P* < 0.001.
